# The challenge of compassion in predator conservation

**DOI:** 10.3389/fpsyg.2022.977703

**Published:** 2022-08-25

**Authors:** Simon Pooley

**Affiliations:** ^1^Department of Geography, Birkbeck, University of London, London, United Kingdom; ^2^School of Life Sciences, University of KwaZulu-Natal, Scottsville, South Africa

**Keywords:** human-wildlife conflict, coexistence, predators, crocodiles, compassion, ethics

## Abstract

This paper argues that compassion for wild animals and the humans living alongside them should be integral to wildlife conservation. Nowhere is this more apparent than in predator conservation, and case studies are used to explore the consequences of wild animal attacks for human victims. Some arguments for extending compassionate consideration to animals seen as individuals are considered, along with the challenges these pose for predator conservation. A way forward from this apparent impasse is suggested, drawing on the capacity approach to embrace human with animal actors. The paper concludes with implications for predator conservation and recommendations, including incident responses sensitive to the traumatic impacts of attacks, and more collaborative approaches to handling human-wildlife interactions taking account of the capacities of local humans and wildlife.

## Introduction

Consideration of the impacts of conserving wildlife on locals has emerged into the mainstream in conservation since the 1990s in the case of (in particular) indigenous peoples (Posey, 1999), and others sharing landscapes with wildlife. In the case of individual wild animals, this emerges into conservation specifically in the 2010s (e.g., Paquet and Darimont, 2010; Wallach et al., 2018), with deeper roots in animal rights thinking (Singer 1975) and what are loosely termed “animal studies” and “human-animal studies” (Echeverri et al., 2018). In this paper, I will address the need for compassion in conservation science through the lens of human-wildlife conflicts and coexistence, specifically. I will first draw on case studies from my research on human-crocodilian interactions to demonstrate the challenges of conserving dangerous animals, in particular for those who must share landscapes with them, and reflect on the role of the conservationist and the need for compassion for victims of wildlife attacks. I will then consider the awkward question of whether animals or humans should be prioritized for compassion in conservation. And if animals should be accorded respect and ethical consideration, how can this be done without paralyzing conservation action? I will conclude with the recommendation that ethical conservation research, policy and practice requires thinking of human-animal communities in interaction: compassion for animals with humans, not animals or humans.

### Background

Working to conserve large predators like the Nile crocodile (*Crocodylus niloticus*) and the mugger crocodile (*C. palustris*) brings responsibilities. Successful conservation of these species, which are seldom confined to protected areas, brings consequences for those sharing landscapes with them, and for both of these species across their extensive ranges, far more habitat lies outside of protected areas than within (Choudhury and de Silva, 2013; Isberg et al., 2019). To date conservationists have responded to this kind of challenge through a focus on managing negative interactions, and providing incentives to tolerate them–in the case of crocodilians, though providing economic benefits through commercial farming and ranching, and ecotourism, and through education about the ecological importance of crocodilians. A toolkit of strategies for mitigating crocodile attacks on humans and livestock has been developed (CSG, 2022a,b).

The human dimensions of conflicts involving wildlife have emerged as a major conservation concern since the 2010s. This includes explicitly addressing the indirect impacts (e.g., fear, opportunity costs) of living with wildlife for locals (Barua et al., 2013). The IUCN initiated a Task Force on Human-Wildlife Conflict in 2016, which evolved into a permanent Specialist Group on Human-Wildlife Conflict and Coexistence in 2022. Research on coexistence focuses on the experiences and perspectives of those sharing landscapes with wildlife outside of protected areas (Pooley et al., 2022).

There has been some work on the social and cultural dimensions of human-crocodilian relations (e.g., Pooley, 2016; Brackhane et al., 2019). However, as is the case for many large predators, there has been little attention focused on the personal and social impacts of traumatic encounters with crocodilians. One exception is Chowdhury et al. (2013) study of what they call Post Traumatic Eco-Stress Disorder in India's Sundarban Delta, including attacks by tigers, sharks and crocodiles.

While I don't wish to develop a conceptual framework for thinking through compassion in conservation in this short piece, I feel that, particularly for consideration of specific challenges around the treatment of animals, Amartya Sen's capabilities approach as developed by Martha Nussbaum provides a useful framework. When considering humans impacted upon by wildlife, this provides the possibility of avoiding cultural relativism on the one hand, and a dictatorial approach insisting on universal rights and values on the other (as IPBES have found, the latter is both unethical and impractical; IPBES, 2019).

Nussbaum's approach is predicated on the idea that all humans should have the freedom to achieve wellbeing. There are ten capabilities (possibilities of functioning people have a realistic possibility of achieving) which all humans should have the right to fulfill, should they choose to. These include life, bodily health and integrity, the ability to sense, imagine and think, feel a range of emotions including love, grief and anger, apply practical reason and reflect on life, affiliate with whom one chooses, have concern for nature and other species, play, and have control over one's environment (Robyns and Byskov, 2020). An ethical approach to conservation, in this view, requires consideration of whether policies and interventions will enable or impinge on these capabilities.

### Shifting from studying victims as data, to engaging with victims' experiences

My research trajectory began with building a long-term database of crocodile attacks in South Africa and eSwatini, to look for patterns and causal links emerging from the aggregated data (Pooley et al., 2019). While doing so I read media stories written at the time of the attacks, and became very aware of the traumatic nature of such attacks. This led me to wonder what the longer term consequences were. The only obvious way to investigate this was to travel to meet victims or their families and friends, which I began to do in the early 2010s.

It was obvious that such encounters must be very sensitively approached and conducted. I was aware that for many attack survivors, or the relatives of those who were killed, the story–as presented in the media, and “resolved” by conservationists through dealing with the problem crocodile or where available providing compensation–was not over. Many had either lost someone close to them, of great importance in their personal lives, with emotional and social and often economic consequences, or suffered life-changing injuries.

If I was advocating the survival of crocodilians in the habitats which they shared (and increasingly share, as climate change, demographic change and land conversion for agriculture and other uses pushes people into formerly uninhabited wetlands) with humans and their livestock, then I needed to understand the consequences for those on the “sharp end” of crocodilian conservation. I began by researching media reports and literature in attacks in more detail, but then by tracking down and interviewing victims in South Africa and eSwatini.

Then, in 2019, I was hosted by Anirudhkumar Vasava and Dhaval Patel of the Vidyanagar Nature Conservancy (VNC) in Gujarat India, to explore human-mugger conflict and coexistence (see Vasava et al., 2015). We traveled around the Charotar region (in Anand and Kheda districts) with Vishal Mistry, Niyati Patel and VNC colleagues, and then in Vadodara District with mugger expert Dr Raju Vyas. In this exploratory visit, we traveled to 19 villages and numerous wetlands to speak with victims of mugger attacks (Pooley et al., 2020).

In this paper I will choose just seven case studies (see Supplementary material for a table of interviews) from these travels and interviews, to illustrate the range of consequences traumatic encounters with predators can have, and highlight the need for compassion in conservation. These are the stories of poor rural people after the sensational event of their being attacked, and the consequences for their lives after the media and the authorities have lost interest in them. My purposes in talking to them were fully explained, and informed consent obtained. All of those discussed here wanted their stories told, and did not want anonymity. My argument is that conservationists must never lose interest in them. Further, I would urge psychologists and others to focus far more interest on studying trauma including PTSD in the victims of attacks by wild animals.

## Case studies

### Sihle Sibonelo Hlatjwako

On 28 March, 2018, I interviewed Sihle Sibonelo Hlatjwako at her homestead near the Mbuluzi River, north of Simunye in northeastern eSwatini (formerly Swaziland). Sihle was then an 18-year-old schoolgirl, in her penultimate year of school. In January 2018, Sihle had been washing clothes in the river below the homestead, when a crocodile seized by her by the wrist and pulled her into the river. She struggled, being submerged and re-surfacing five times, before managing to grab onto some reeds and scream for help. She was rescued by two brave teenage boys. Although game reserve staff were quickly on hand, the family waited for the police to fetch Sihle and take her to hospital. It was explained that those who can't afford to pay only get free treatment if the police bring you to hospital.

Sihle was in some sense lucky to escape with her life, and only damage to her wrist. However, months after the attack, the skin graft was very visible, and she had not recovered full use of her left hand. The Nkomo family, with whom she lives, are very poor and couldn't afford to both pay school fees and physio treatment to help her regain use of her hand. They therefore decided to pay for her treatment, and took her out of school. Sihle is a bright girl, and once her shyness had worn off, we spoke in English. Remarkably, she bore the crocodile no ill will, and regarded the attack as an unfortunate accident. She was very interested in learning about crocodiles, and I gave her a booklet and poster I have developed.

The point of this case study is, primarily, that this brave girl will almost certainly never complete school. Of passing interest to the press, the attack was regarded as “minor” and there is no compensation paid for such attacks. For a poor girl from a rural background, this event has seriously limited her future life possibilities, and leaves her with a partial disability. It is unsurprising that locals resent the presence of crocodiles which are protected animals, dislike the lack of control over their environment in not being able to deal with crocodiles, and hold conservationists responsible for preventing attacks.

### Vikram Gohil

On 27 September 2019, I interviewed a young man named Vikram Gohil on the edge of the wetland of Deva village, Anand District, Gujarat State in India. Almost exactly a year previously, he had swum out into the pond to help one of his water buffalo which had become entangled in water hyacinth. He was neck-deep when a mugger seized him by the shoulder. He struggled, but it wouldn't let go, and another two small mugger also approached, though did no damage beyond scratches. He managed to struggle to shore, where the mugger left him.

Vikram belongs to the Waghri community, and though he received treatment at the government hospitals in Deva and Vasol, he claims that it was traditional medicine (monitor lizard fat) that healed him. While physically, the only traces of Vikram's encounter are scars, he is clearly still psychologically troubled by the attack. He is now afraid of the water, and believes that the mugger, having tasted his blood, will attack him if he enters the pond. At the same time, he doesn't believe harm should come to the mugger, and suggests it may have been a mother defending young.

In discussing the incident, he kept repeating how frightened he had been, and eventually admitted that while he has no bad dreams, he still experiences “moments of fear.”

### Ratilal Vasava

Another victim of a mugger attack, Ratilal Vasava from Pingal Wada Village in Vadodara District, Gujarat, was pulled into the Dhadhar River by a mugger while tending his cattle. He managed to fight free, without serious injury. However, when we visited he was taking antibiotics to cope with the serious infections that often result from crocodile bites, and he is now fearful of the river he has lived next to all his life. Like Vikram, Ratilal now also experiences “moments of fear,” which he described with the Gujarati term “*bhankara*” meaning something like an illusion that spontaneously comes to you.

### Vinu Vasava

We interviewed Vinu Vasava in Pingal Wada village. Her husband Radha was killed by a mugger, and she said she was in shock for 3 months afterwards. She is a mother of five daughters, and having lost her husband, who was the breadwinner, had to become an agricultural laborer. She hadn't heard about compensation, and didn't receive any. Her life has changed fundamentally, as she must work to support her family and pay for the marriages of her four unmarried daughters. As a devotee of the Hindu goddess Khodiyar, always shown astride a mugger crocodile, she bears no ill will to mugger crocodiles.

### Madhuben Naran Vasava

In Mahadev village, Vadodara District, we interviewed Madhuben Naran Vasava. This petite, elderly widow had been bitten on the left arm by a mugger while washing clothes 5 years previously. Three months of treatments and a skin graft left her with an emaciated, scarred arm which doesn't fully function. Her compensation covered a fifth of the cost of her treatment, forcing her to mortgage her agricultural land, and she is still paying it off. When asked how she felt about the mugger, she replied that she knew she couldn't express her feelings, because that would be illegal. She thought the Forest Department should trap and remove the mugger from the river.

### Kalapn Rana

We interviewed Kalapn Rana at her home near Goraj, in eastern Vadodara District. Several years previously, the family had enjoyed a picnic on the banks of the Dhadhar River. It was very hot, and after the others left, Kalapn and her mother-in-law Manhar took a quick swim. Afterwards Kalapn was wringing out her petticoat when a mugger seized her by the left hand and pulled her into the water. Manhar jumped into the river and a tug of war ensued, the mugger dragging both across to the opposite bank. Amazingly, Manhar managed to drag Kalapn back across the river. Two men then came to the rescue, forcing the mugger to release Kalapn and retreat into the river.

**Figure 1:**
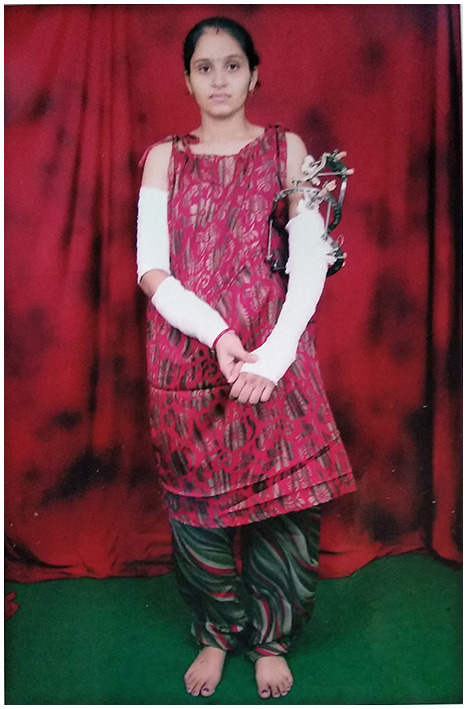
Kalapn Rana during her treatment following a crocodile attack.

Kalapn by this time had been bitten several times on the left forearm, shredding muscles and splintering bones. Fortunately, the family are reasonably well off, as she had to then endure 9 operations to repair her arm (see Figure 1). As she was 2 months pregnant, and required anesthesia and radiation, they had to abort the fetus.

For 3 months, Kalapn dreamed of being taken by the mugger [(Chowdhury et al., 2013) note one case of bad dreams following a crocodile attack], and it took 2.5 years to recover from her injuries. However, she still has fixed flexion disability, and this young mother (she has a baby now) can't comb her own hair. At the time of the attack, she had recently graduated and had intended to continue her academic studies (which clearly was the expectation of her mother-in-law), but she had to give this up. Kalapn now avoids the river completely, and can't swim in a pool without checking for mugger first. The Forest Department paid compensation (a tenth of the cost of her treatment), and tried but failed to catch the mugger, which is still living locally.

#### Compassion for victims

There is something important to be said about fatalities in animal attacks. This is the outrageous assertion that rural folks in remote communities somehow value life more “cheaply” and are in some way “philosophical” about losing husbands, wives, children, friends. This perception was voiced in a comment on a story on crocodile attacks I published in *The Conversation* (Pooley and Marchini, 2020). I can assure anyone who might share this view that (for example) the parents I interviewed, some several years after an attack, were still devastated by the loss of their child.

Finally, it should be noted that asking people to share their experiences is asking a lot. It requires respectful and sympathetic attention, and great care and tact in questioning (Pooley et al., 2020). Many relatives of victims show me photographs of their loved ones after attacks. It seems impossibly grim that they cling to this evidence of their final memories of their loved ones. For the conservationist, absorbing relatives' grief, and witnessing the often horrific images of damaged corpses, also takes a toll. I feel it is a necessary one, particularly where conservationists and supporters of conservation live far removed from the consequences of their successful efforts to conserve dangerous wildlife.

### What should be the focus of compassion in conservation, animals or people?

When it comes to compassion in conservation, there is an awkward fracture between human-focused and animal-focused rights orientations. In an epoch of crashing biodiversity, almost entirely due to anthropogenic impacts, this is not an easy dichotomy to address (e.g., Pooley and Redpath, 2018; Vucetich et al., 2018). Supporters of Compassionate Conservation (Wallach et al., 2020) criticize conservation for justifying harm to sentient animals through instrumentalism (the animal as means to an end), holism or collectivism (species are more important than individuals) and nativism (human-assisted species are unnatural). They argue that in fact all sentient beings are persons, as recognized in some non-Western traditions, and thus worthy of respect and compassion. It is not sufficient for them that “ethical concern for individual animals [forms] an important element in conservation best practices” (as argued by Hayward et al., 2019), if that is subordinated to landscape-level biodiversity protection concerns. They argue that conservation's prioritization of “native” and “wild” animals is misplaced and shouldn't justify violence to either wild or feral or domesticated non-human animals. Compassion, in this view, links us ethically to *all* non-human “persons” equally (Wallach et al., 2020).

Just as animal rights pressure on conservationists has improved the care of captive wild animals, for example, so compassionate conservation's focus on the fates of individual animals can be a corrective to indifference to them where that exists. Further, widening consideration of what is valuable beyond ideas about “pristine” or “wild” landscapes, and only native wild animals, is valuable on our fast-changing, human-dominated planet. However, there remain key challenges to how compassionate conservation works out on the ground, particularly pertinent for those managing potentially dangerous animals like crocodiles. For example, what of the rights of local people living with dangerous wildlife not to like or want that wildlife around, and their right to be angry about depredations on their communities and to dislike individual animals for their actions (Smith, 2020). How do we work in landscapes where some locals revere and respect particular species of wildlife, while others see them as a dangerous threat, or where locals identify particular problematic individuals as were-animals, different to “normal” animals (Pooley, 2016)?

If compassion requires consideration of all the actors involved in human-wildlife interactions, and humane actions including (if, as a last resort, required) lethal control (humanely performed), then that seems implementable. If it means that every individual has an inviolable right to life, including for example an introduced predator threatening the existence of a native species, or a crocodile threatening the lives of local farmers, then that does not. While trapping and removal of a problem animal may be preferable, it isn't always possible, or not in time to prevent disaster. Finally, if we follow rights approaches to logical conclusions, there are awkward questions around the assumed rights of prey not to be predated upon (Keulartz, 2016).

### The capability approach for non-human animals

How else, then, might compassion for non-human animals be integrated into conservation? How can we include animals in an ethical conservation without being limited to observing the inviolable rights of each individual animal? How might we be empowered to act compassionately and proactively, rather than get stuck on what we cannot do? How can the need of certain animals to attack and eat others be acknowledged in framework that grants them all ethical consideration?

I find Nussbaum (2006; as discussed in Bendik-Keymer, 2014) capabilities approach to a more-than-human ethics interesting here, as a basis for ethical consideration of non-human animals not confined to proscriptions on the treatment of individual animals. Nussbaum suggests that if humans can feel wonder looking at nature or a complex organism, then that suggests it is good and right for it to flourish and persist as the kind of thing it is. It is, then, a being worthy of respect, and so is its striving to persist and flourish. So much for extending the circle of ethical consideration beyond humans. When it comes to what should guide ethical action, it is *empathy* which guides us to which beings worthy of wonder deserve justice (not all do, in this interpretation). If we can imagine other beings as having a stake in their own existence, and hence a capacity to be wronged (thwarted), then those beings have a claim to justice.

The capacity of charismatic mammals like elephants to experience social and individual trauma and PTSD-like symptoms has been recognized (Bradshaw et al., 2005; Münster, 2016), and is an interesting area of research, albeit proscribed by human sympathies in terms of species (though see Zanette and Clinch, 2020), as is the capacity of wild animals for compassion. Apparent tolerance of “habituated” animals for humans, in situations where they must share landscapes, may mask heightened stress levels with adverse effects on individuals and populations (Whittaker and Knight, 1998; Bejder et al., 2009). Of course, the same goes for humans living alongside dangerous wildlife.

Nussbaum's approach attaches moral significance to species, and not solely to individuals (Keulartz, 2016). In an age of what some call ecocide (e.g., Posey, 1999), this extends moral consideration beyond the individual to the species, and by extension (in terms of their needs) to habitat.

If we look beyond solely what *shouldn't* be done to individual animals, which is the focus of much animal rights work, we then can also consider what a species-specific norm of flourishing might look like. That is, what is the appropriate benchmark for judging if a member of a species has the necessary opportunities to flourish (fulfill its capabilities). Humans have a proactive positive role to play here, a duty to support the capabilities of other beings (how widely that circle is drawn is another matter), up to a minimum threshold level calibrated for their species. Flourishing is, after all, about more than just the absence of pain or discomfort. This approach to ethics also allows for a coherent approach to interventions where it is not possible, due to external circumstances like habitat destruction, to allow wild animals to continue with their lives free of human influence (Keulartz, 2016). It need not be limited by notions of wildness or pristineness, either.

### Beyond compassion for humans, or compassion for animals

Humans and wildlife have shared landscapes for millennia, and even in the partitioned lands of North America and Western Europe, factors like urbanization, land abandonment and climate change are increasingly forcing them to cohabit rural and even urban landscapes (König et al., 2020). There are a diversity of ways of understanding and dealing with the resulting situations, shaped by particular cultures, knowledge systems, traditions of using and interacting with the land, histories of interaction, and the particular ecological contexts and fauna (species, individuals, communities). Different sets of humans and animals (individuals, species, perhaps cultures) have learned to interact in particular, mutually influenced ways. The point is, it is nonsensical to consider compassion in conservation without thinking in terms of the communities of humans and non-humans where interactions and their consequences unfold.

So, if a capabilities approach were to be developed, there is collaborative context-specific work to be done in deciding on which capabilities particular species or other groupings of animals typically have, as a non-anthropocentric yardstick for determining how to ethically interact with them (Keulartz, 2016). Doing so requires acknowledging that species concepts, and taxonomies of species (including cultural ones), vary. Then there is consideration of communities of humans and species of wildlife in interaction in particular contexts, sharing spaces and resources. There will be important cultural dimensions to how we understand all this, and we have much to learn from indigenous peoples and locals who coexist with wildlife.

The context-specificity of human-wildlife conflict and coexistence means that there is unlikely to be a moral standard with 10 inalienable rights commandments applicable everywhere for all peoples and species. This doesn't mean it isn't possible to formulate principles relating to what should be considered when attempting to show compassion in conservation, or to understand coexistence where it occurs (and not mess it up with tone-deaf interventions), and to foster compassion and coexistence where they don't exist. It remains to be seen whether sets of capabilities could be formulated for agreed groupings of non-human beings. It is an intriguing possibility that, working with locals, it may be possible to formulate more precise versions for particular landscapes. These would need to be informed by both ecological and cultural understanding of individuals and types of particular non-human beings, and communities of interaction.

## Conclusion

I want to preface my conclusions with my final case study, the story of the Ode family. I spoke with Hemant Ode, a lean 42-year-old farmer and laborer, sitting on a wooden frame bed under a lean-to outside his home near the edge of Traj Village pond, in Kheda District, Gujarat. Those present included his wife Naniben, and Anirudhkumar Vasava (translating). Hemant told us how his only daughter, Hetal, had been seized and drowned by a mugger in the pond nearby while washing a big steel pot. She was only 9 years old. Both parents were clearly still devastated, and a framed portrait with the girl's necklace looped around it hangs on the front of their home. My own daughter was nine at the time of our interview, and afterwards I reflected on the despair and rage I would have felt, should this tragedy have befallen me and my family. Would I have continued to tolerate crocodiles living in the pond that we had to use daily for water?

Hemant Ode is now a “mugger mitra” (crocodile friend), who dedicates time to educating others on how to live more safely alongside crocodiles. In addition, he participates in rescues, that is, safe capture and removal of mugger found in areas where harm may ensue for people, or crocodiles. Remarkably, during my visit to Gujarat, he was involved in the safe capture and removal of a crocodile that was very possibly the one which had killed his daughter, and later an elderly man, in Traj Village pond. It had been removed to a nearby wetland, Pariyej, and was trying to return home to Traj pond (pers. comm. Vishal Mistry).

There is much to be learned about the motivations of someone like Hemant. For instance, in what ways is his compassionate response to a tragic event influenced by his cultural context, his personal beliefs, and his individual life history and experiences at the pond? Is his response a culturally mediated or perhaps more universal psychological coping mechanism for reframing a tragic event? There is much to learn about different cultures' mechanisms for coping with trauma, and specifically how particular cultures' explanations of the causality of animal attacks may provide adaptive ways of coping with this trauma, and enable coexistence with dangerous animals (Wilson, 2007; Pooley et al., 2020).

There is also much to be learned from the behavior of the mugger that share ponds, mostly peaceably, with humans in the wetlands of Gujarat, and perhaps from comparative study of where relations are hostile, in the same region. That is, rather than continue to consider humans and wild animals separately in ethical terms, and to study their behavior separately, we need to study human-wildlife communities and their interactions in more holistic and interdisciplinary ways.

For the conservationist, there are many levels at which compassionate consideration is required here: for the parents of a girl killed by a mugger; for locals living alongside mugger, with varying exposure to, and beliefs about mugger; for individual mugger crocodiles sharing habitat with humans and livestock; and for the challenges mugger crocodiles face in the agricultural landscapes of central Gujarat.

### Some implications

Conservationists involved in conserving dangerous wildlife have an ethical duty to engage with the consequences for local people living with these animals. Compassion for the victims of encounters is vital, not just for the plight of wildlife in shared landscapes with humans, though this matters too. This requires personal engagement with individuals, not statistical analyses and solely instrumental or economic responses. There is much to learn about long-term consequences of life-changing encounters with dangerous wild animals, and what assistance can then be offered. There is also much to be learned from those who, like Hemant Ode, have found ways to surmount personal tragedies and work to facilitate coexistence with dangerous wild animals.

Some of the lessons may be cultural, emerging from societies with long experience of coexisting with dangerous wildlife; some may emerge from psychological study of how people cope with traumatic encounters; others from the study of animals' traumatic experiences and interactions with humans. Some will be very context-specific, while some may be portable and provide means of fostering coexistence elsewhere. Conservation researchers would benefit from working with psychologists and anthropologists when attempting such studies.

On a practical level, it seems advisable that conservation authorities shouldn't respond to traumatic incidents by sending in individuals untrained in the sensitivities of interacting with traumatized victims and their families, tasked only with performing essentially a law-enforcement function. Adjudicating on whether material compensation is warranted is not, in the first instance, the appropriate response. Training for first responders, and the involvement of professionals with context-specific knowledge and skills in handling traumatic situations and interacting with victims, would improve victims' experiences and relations between communities and organisations responsible for wildlife.

Responses to traumatic encounters with wildlife should also go beyond incident-response, to consider the longer-term consequences for individual humans, and wild animals, and their communities. The consequences for all actors, human and non-human, in terms of their capacity to flourish should be considered when deciding on interventions. The capability to live a full life should be considered, for all actors, but on occasion managers will have to weigh the threats to life posed by dangerous animals against their right to persist. They need to consider the consequences of sparing the life of a dangerous individual, in light of how it may impact on locals, and how it may influence local societies' tolerance for the species. If it cannot be safely and timeously removed to a secure place, that individual animal may have to be humanely killed. Let us also reserve some compassion for those who must take and enact such difficult decisions.

## Data availability statement

The raw data supporting the conclusions of this article will be made available by the author, without undue reservation.

## Ethics statement

The studies involving human participants were reviewed and approved by the School of Social Sciences History and Philosophy Ethics Committee, Birkbeck University of London. The participants provided their written or verbal informed consent to participate in this study. Informed consent was obtained from the individual for the publication of any identifiable images or data included in this article.

## Author contributions

The author confirms being the sole contributor of this work and has approved it for publication.

## Funding

This research was funded by a Birkbeck/Wellcome Trust Institutional Strategic Support Fund Grant.

## Conflict of interest

The author declares that the research was conducted in the absence of any commercial or financial relationships that could be construed as a potential conflict of interest.

## Publisher's note

All claims expressed in this article are solely those of the authors and do not necessarily represent those of their affiliated organizations, or those of the publisher, the editors and the reviewers. Any product that may be evaluated in this article, or claim that may be made by its manufacturer, is not guaranteed or endorsed by the publisher.
