# Supplementary material for: The challenge of compassion in predator conservation
Source: Front Psychol. 2022 Aug 25;13:977703. doi: 10.3389/fpsyg.2022.977703 (PMC9454015; doi:10.3389/fpsyg.2022.977703)
Supplement: Supplementary file 1 [file Table_1.DOCX]

**The Challenge of Compassion for Predator Conservation**

**Supplementary** Information

**Interviewees in order of mention in the text**

| Date | Interviewee details | Key participants | Location |
| --- | --- | --- | --- |
| 28 March 2018 | Sihle Sibonelo Hlatjwako, female, survivor of a Nile crocodile attack | Sihle, Sharp Shabangu (translation), Simon Pooley, mother and eldest sister of Sihle | Near Simunye, northeastern eSwatini |
| 27 September 2019 | Vikram Gohil, male, survivor of a mugger attack | Vikram, Anirudhkumar Vasava (translation), Vishal Mistry, Simon Pooley, Vikram’s cousin Gopal Gohil, and friends. | Next to Deva Village wetland, Anand District, Gujarat, India |
| 28 September 2019 | Ratilal Vasava, male, survivor of a mugger attack | Ratilal, Anirudhkumar Vasava (translation), Raju Vyas, Simon Pooley, family members | Pingal Wada Village, Vadodara District, Gujarat, India |
| 28 September 2019 | Vinu Vasava, wife of Radha Vasava who was killed by a mugger | Vinu, Vaja Vasava, two women not identified, | Pingal Wada Village, Vadodara District, Gujarat, India |
| 29 September 2019 | Madhuben Naran Vasava, female, survivor of a mugger attack | Madhuben, the wife of her deceased husband’s younger brother, Raju Vyas, Simon Pooley, Vishal Mistry | Mahadev Village, Vadodara District |
| 29 September 2019 | Kalapn Rana, female, survivor of a mugger attack | Kalapn and Pruthraj (husband), mother-in-law Manhar, Raju Vyas, Tushar Vaishnav, Vishal Mistry, Simon Pooley | Her home near Goraj, Vadodara District |
| 25 September 2019 | Hemant Ode, male, father of fatal attack victim Hetal Ode | Hemant and Namiben Ode, Anirudhkumar Vasava (translation), Niyati Patel, Vishal Mistry, Simon Pooley | Their home next to Traj Pond, Kheda District, Gujarat |
